# Institutional care and education: circulation of knowledge about epilepsy in Sweden 1915–40

**DOI:** 10.1017/mdh.2024.23

**Published:** 2024-07

**Authors:** Johanna Ringarp

**Affiliations:** Department of Education, Uppsala University, Sweden

**Keywords:** Epilepsy, Sweden, Circulation of knowledge, Children, Institutions, educable

## Abstract

This article focuses on the circulation of knowledge about epilepsy in Sweden between 1915 and 1940. During the period medical research on epilepsy increased, which simultaneously brought a new degree of specialisation and distinction between branches of medicine. The aim of this article is to study the impact of new medical knowledge about epilepsy on the treatment and education of children with epilepsy in Sweden. In order to concretise the aim, the study focuses on the asylum Margarethahemmet. The key source material consists of Margarethahemmet’s annual reports and yearbooks. The minutes of the meetings of the Swedish General Association for the Care of the Feebleminded and Epileptic for the period 1915–1938 have been used as supplementary material. In order to trace the impact of medical discoveries on Margarethahemmet’s operations, contemporary scientific articles, mostly from Germany, have also been used. The article demonstrates how new research and new knowledge was sought internationally and nationally, to provide doctors and special teachers at the asylum with a proper knowledge about education, care and treatment for children with epilepsy. The increased understanding of the disease directly impacted the ability of a stigmatized group – people with epileptic disorder – to actively participate in society on the same terms as others.

## Introduction

The first half of the twentieth century saw an increase in medical research into epilepsy, with a new degree of specialisation and separation between different branches of medicine. Neurology and neurosurgery became separate fields, increasingly remote from psychiatry. New theories and new types of practical knowledge about epilepsy emerged, resulting in a new approach from both scientists and clinicians.^1^ Thus, the question of which forms of knowledge were considered relevant in a clinical setting becomes central. The new medical knowledge about epilepsy also paved the way for a new approach to educational opportunities for children with epilepsy. The aim of this article is to study the impact of new medical knowledge about epilepsy on the treatment and education of children with epilepsy in Sweden in the first half of the twentieth century. To concretise the aim, the study focuses on the epilepsy home Margarethahemmet and its medical inspector Henry Marcus. The questions that will guide the study are as follows: In what ways did new medical findings guide Margarethahemmet’s operational and educational focus? What type of care and treatment methods were used? The period under investigation extends from the opening of the epilepsy home in Knivsta, outside Uppsala in 1915, until the resignation of Henry Marcus as the home’s attendant doctor.^2^ Explanations will be sought by studying the circulation of both medical and educational knowledge.

## Sources, methods, and theories

The key source material for this study consists of Margarethahemmet’s annual reports and yearbooks. They provide detailed data on the number of children admitted, where they came from, their needs, and their educational opportunities. The minutes of the meetings of *Allmänna Svenska Föreningen för Vården om Sinnesslöa och Fallandesjuka* (the Swedish General Association for the Care of the Feeble-minded and Epileptic) for the period 1915–38, as well as lecture notes from Thorborg Rappe, the director of the *Slagsta skolhem* (Slagsta Residential School), have been used as supplementary material, providing a useful insight into what the Swedish experts and those working at residential facilities believed were the key issues. To trace the impact of medical discoveries on Margarethahemmet’s operations, contemporary scientific articles, mostly from Germany, have also been used.

Methodologically, content analysis has been used to identify common themes in the divergent types of source material (annual reports and scientific articles).^3^ Through repeated readings of the source material, the content and text passages that address issues of practical treatment as well as theoretical knowledge about epilepsy have been selected. In addition to the Margarethahemmet’s annual reports and yearbooks, there is also an anniversary book that was published when the asylum turned 50 years of age. As for Henry Marcus, I have found no surviving documents written by him except for the articles he wrote in Swedish and those in international journals.

Theoretically, the article draws on the growing field of history of knowledge.^4^ In this context, the concept of circulation of knowledge will be used as an analytical tool. Historians Johan Östling and David Larsson Heidenblad describe circulation of knowledge as a catchword that ‘seems to be able to denote practically any form of movement or process…’.^5^ In Sweden, however, researchers have only recently applied it in a more synthesised way, not least to shift attention from the production and origin of knowledge to studying its circulation and effects, i.e. allowing questions of societal relevance and reach to be at the forefront of investigation.^6^ According to the historian Philipp Sarasin, knowledge depends on circulation to ‘work’. It reacts to ‘impulses’ from other fields of knowledge and different social spaces, is taken up again in other places, and is transformed in the process.^7^ In particular, the paper will focus on what has been considered legitimate knowledge for the treatment of epilepsy and what kind of teaching could be provided in the institutions.

I would like to preface the analysis section with some comments on the terminology used, including epileptic, falling sickness, and feeble-mindedness. During the period being studied, such terms were commonly used in Swedish and many other languages. Many of the Swedish terms derived from the system of diagnosis of insanity developed in the early 1860s by Nils Gustaf Kjellberg, a professor of psychiatry at Uppsala University.^8^ The terminology, as the education specialist Thomas Barow puts it, ‘evolved in a certain social and societal context’.^9^ It was primarily doctors and educators who had the prerogative of interpretation when it came to such categorisations. To reduce the stigma surrounding the disease, it has been suggested in recent years that the word ‘epileptic’ be replaced with ‘people with epilepsy’.^10^ Because this article is about societies and doctors’ views on the care and treatment of epilepsy in the early 1900s, I have used much of the language of the time, well aware of the historical ramifications.^11^

The article starts with a summary of the existing research, an introduction to the different classification systems of epilepsy during the period, and a brief history of the epilepsy asylum Margarethahemmet. The analytical section that follows is divided into four sections: *Teaching the educable and the uneducable* discusses what kind of education the children with epilepsy at Margarethahemmet received, what type of teacher training the educator had, and how this medical-pedagogical development was part of a larger movement of ideas. Sections two and three (*Seizures and their consequences* and *Antiepileptics and new diagnostic methods)* deal with the medical treatment of children with epilepsy and the progress made during the period in focus. In the fourth section, *Knowledge production about epilepsy and its causes*, I return to Margarethahemmet and discuss the medical inspector’s expectations that new expertise and knowledge about surgery would help the residents. Finally, I present the results of the study and the impact that I believe the circulation of knowledge has had on the care, treatment, and education of the children at the epileptic asylum.

## The history of epilepsy

Owsei Temkin and Walter Friedlander have written useful overviews of the history of epilepsy in the Western world, from classical times to the early twentieth century. Both focus on the advances in medical science in their broader societal context, including legislation, regulation, and the opportunities for education and housing of people with epilepsy.^12^ International studies of the history of psychiatry, neurology, and eugenics also frequently touch on epilepsy.^13^ The main studies on the history of epilepsy have been produced by neurologists, and thus rarely correspond to the standards of historical methodology and theory.^14^

In Sweden, too, the history of epilepsy has mainly been written by medical doctors.^15^ Exceptions include studies by the historian Mattias Tydén and the historian of ideas Gunnar Broberg on scientific racism and sterilisation policy in Sweden, which touch on the handling of heredity and forced sterilisations of people with epilepsy.^16^

Epilepsy was defined as a psychiatric disorder well into the twentieth century and was thus discussed in numerous psychiatric and medical studies. In recent years, the care and treatment of other groups in Sweden, including people with epilepsy such as the patients at *Vipeholms sjukhus* (Vipeholm Hospital) – an asylum for so-called feeble-minded in the south of Sweden – have been the subject of some interest.^17^ However, those studies have not focused on the treatment, education, and care required for people with epilepsy.

Research on epileptic homes is even less well studied. Jona T. Garz has investigated how feeble-minded and epileptic children were treated at the asylum Berlin-Dallendorf in Germany at the end of the nineteenth century. Garz also explores children’s educational opportunities and shows the conflict that arose between those who believed that feeble-minded children should receive some form of education in an institution and those who believed that they should attend remedial classes.^18^ In another study of the epilepsy institute in Wuhlgarten near Biesdorf, Germany, Ulrich Beyer has shown that new medical knowledge after the First World War led to the separation of the diseases ‘insanity’ and the care of ‘epileptics’, which in time led to care being designed specifically for the patients.^19^ There are also some more descriptive studies of the foundation of the epilepsy homes Bethel (Germany) and Filadelfia (Denmark) during the late nineteenth century. These histories show that it was mostly philanthropic activities that took care of people with epilepsy before care and treatment for epilepsy became part of the general health care system. Both Bethel and Filadelfia have since changed character, from being known as asylums to becoming major epilepsy research centres.^20^

Thus, the history of epilepsy homes in Sweden has to date only received limited attention. Lena Lennerhed has discussed in an article how the patients at Stora Sköndal in the late 1970s viewed themselves and their illness. Stora Sköndal, in the southern part of Stockholm, was Sweden’s largest asylum for ‘epileptics’, where mainly adults with epilepsy lived from 1902 until it was closed in 1980.^21^ Through a case study of Margarethahemmet, this article sets out to shed greater light on the views of medical science on epilepsy and the treatment of the condition in Sweden. Additionally, the present article discusses how the knowledge of new treatment methods influenced the discussion about the ability of ‘epileptics’ to be educable.

## Classification and differentiation

Epilepsy, or falling sickness as it used to be called, is a condition long recognised, discussed, and mythologised. There have been many theories about the causes of epilepsy, and people with epilepsy were described as having ‘ixophrenic’ traits, a supposed personality marked by ‘viscosity’ or ‘persistence’, trapped in random patterns of speech or thought. Equally often, epilepsy was associated with demonic possession or divine punishment – the gods speaking through the sick. The idea that ‘epileptics’ were ‘abnormal’ rather than sick determined how the condition was viewed and treated in the past.^22^ It is also striking how unremitting such prejudices have been, despite advances in medical knowledge and treatment methods.^23^ Today, epilepsy is described as a neurological condition that affects the nervous system, and that seizures occur when certain nerve cells in the brain are overactive.^24^ Until the 1950s, epilepsy was seen as closely connected to feeble-mindedness and some inmates in asylums for feeble-minded were also diagnosed with epilepsy. Vice versa, at epileptic colonies, many were judged to be ‘retarded’.^25^ Therefore, inmates were sometimes moved between feeble-minded and epileptic asylums.

Previous research has revealed that the institutional care designed for the feeble-minded in the late nineteenth century focused on providing children with a moral upbringing and saving them from poverty. Initially, funding was mainly provided by philanthropists, but eventually government grants were made available to the institutions.^26^ Economic arguments carried weight in the debate: if the government ensured that ‘educable’ feeble-minded children received an education, they would no longer be a burden on society. During this period, views on childhood were changing, too. Children were increasingly seen as a socioeconomic investment, and society was expected to take greater responsibility for their upbringing – and by extension for the care of feeble-minded children.^27^ Despite society’s growing interest, and investment in children, ‘epileptics’ were long excluded because of beliefs that ‘epileptics’ were deviant or ‘abnormal’, and the poor medical understanding of the disease. Matters were not helped by it being considered difficult to have ‘epileptics’ on the same wards as the feeble-minded, and the institutions for feeble-minded children were already at full capacity, even before ‘epileptics’ were admitted. ‘Epileptics’ who were also considered feeble-minded were therefore particularly vulnerable.^28^ Even when the neurological clinics in Sweden were formed from the 1940s onwards, people with epilepsy were still primarily treated in epilepsy hospitals and institutions. This meant that epilepsy care came later into the new clinic structures than the care of other neurological diseases.^29^

Even though epilepsy was seen as related to feeble-mindedness, there was a difference regarding the care offered. In the 1950s, there were nearly 200 asylums for the feeble-minded in Sweden and between 1900 and 1950, persons admitted to asylums for the feeble-minded increased from 800 to about 13,000.^30^ In comparison, there were only twelve epileptic asylums in operation in Sweden throughout the twentieth century. Two were residential schools for children; the others were institutions for adults. Thus, only a small percentage of the people with epilepsy were admitted to one of these institutions. Most of them, if only because of better medicines in the twentieth century, were able to live in the community like anyone else.^31^ The total number of places in epilepsy hospitals was 972, of which 206 places were for children under the age of sixteen years, 331 were for women, and 435 were for men older than age sixteen.^32^

The medical developments – with regard to both medication and surgery – that led to better treatment for ‘epileptics’ are seen in the article as one of many examples of the circulation of medical knowledge during the period. However, the establishment of medicine as a science in the twentieth century was not only a success story. It also led to what had previously been considered moral failings, such as adjustment difficulties and antisociality, being deemed diagnosable failings in the individual, which could often be explained by heredity. This approach was later developed in hereditary and eugenics.^33^

## Margarethahemmet : a school home for children with falling sickness

‘Epileptics’, especially if also considered feeble-minded, were a vulnerable group, especially because one prominent theory was that many epileptic seizures could lead to feeble-mindedness.^34^ At Margarethahemmet, the question of whether the children were educable or not was important. Daily routines and a calming environment were supposed to guarantee a relatively normal upbringing for the allegedly uneducable children.^35^ Similar ideas about the importance of the environment and daily routines in activating good habits are also found in the well-known medical-pedagogical publications from Èdouard Séguin, Carl Wilhelm Saegert, and Johann Guggenbühl.^36^ The owners of Margarethahemmet, the Association for the Care of Feeble-minded Children with Epilepsy, felt from the outset that it was important for children with epilepsy to receive care and treatment in a quiet environment, but also that the place be close to a major city.^37^ In the early years, the centre was located in the western part of Stockholm, which meant proximity to doctors, pharmacies, and other facilities that were important for the running of the institution.^38^ Despite the favourable location, Margarethahemmet soon outgrew its premises. The association therefore decided to build an entirely new care institution out of town. A property near Lake Valloxen, just over a kilometre from the municipality Knivsta, was chosen, where there was a medical clinic. That the Uppsala University Hospital was only 20 kilometres away was also advantageous.

When the premises in Knivsta were ready for occupancy in 1915, the home was considered to be one of Sweden’s most modern health care facilities, and in 1926 a cold bathhouse with a height-adjustable floor was built to enable residents to bathe by the lake.^39^ The board of Margarethahemmet had also begun plans to expand its activities and allow their ‘protégés’ to remain in the home even after they had finished school.^40^ The reason for this was that the board feared that the clients ‘in the absence of such care, would probably deteriorate again, or at least would find it difficult to sort themselves out on their own’.^41^ On the new property, there was the possibility to build a workhouse, where seventeen female and six male patients could live.^42^ This new strategy – to take care of those with epilepsy who had left school – was well in line with what was being discussed at the same time both by the Swedish General Association for the Care of the Feeble-Minded and Epileptic and in other countries.^43^

## Teaching the educable and the uneducable

Children’s receptivity to education was usually considered to be directly related to their intellectual ability.^44^ In addition, doctors believed that an early onset of epilepsy and more frequent seizures caused the child to become feeble-minded more quickly. Neurologist and psychiatrist Henry Marcus said feeble-mindedness could take two forms, ‘idiocy and imbecility’, both with varying degrees. Idiocy was defined as a ‘state of deepest insanity’, and accordingly any form of education was ruled out.^45^ With the diagnosis ‘imbecility’, a child was regarded as feeble-minded, but could, depending on the severity, possibly be taught in a remedial class.^46^ As of 1897, there were grants available for feeble-minded ‘epileptics’, and from 1915 for ‘educable’ children at one of the epileptic schools.^47^ Margarethahemmet’s annual reports state how many of the children examined were classified as educable and uneducable, respectively. In the Swedish mental health system, the classifications of ‘educable’, ‘uneducable’, and ‘difficult to manage’ were used to assess the care needs of patients during the period. Depending on the classification of the individual patients, they were placed in an institution with the appropriate specialisation.^48^ Of the 38 children at Margarethahemmet who were resident in 1916, 18 were considered educable and 20 uneducable.^49^ By 1924, the number of children had risen to 78, of whom 36 were categorised as educable and 42 uneducable.^50^ Fifteen years on, there were 103 children, of whom 62 were educable and 41 uneducable.^51^

The reason for the increased number of educable does not appear in the minutes, but throughout the period the importance of providing the pupils with clear daily routines is discussed.^52^ And perhaps the daily routines and the calm environment made it easier for the ‘difficult to manage’ to absorb knowledge, i.e. become more educable. In a report from 1930, the Swedish physician Carl Sebardt claimed that it was essential for patients to ‘have their time regularly divided with fixed times for rest, work, and meals’.^53^ Similar ideas about the importance of seclusion and peace in reducing the number of seizures for individual patients circulated in Germany and Denmark, and inspired many of those in Sweden who wanted to improve epileptic care.^54^ In a German context, Barbara Kuhlo has described that the aim of what in German is called *Heilpädagogik* was to enable the feeble-minded person to establish a relationship with the surrounding world and achieve a level of development of the person concerned that is useful in practical life.^55^ And Reverend Van Koetsveld, who first initiated the first Dutch ‘School for Idiots’, talked about ‘Cure by Education’, which meant the inclusion of education and medical aspects in the training programme.^56^

In relation to the classifications educable and uneducable, the question of pedagogy for these children also becomes important to understand. In terms of education of the so-called abnormal it is clear that there were two different schools of thought in Europe during the period: the French medical-pedagogical and the German philanthropic-charitable. In the Swedish context, however, as a result of the study trips undertaken by Swedish teachers and doctors, these two schools of thought were drawn on in concert with one another.^57^ It was a matter of making the inmates medically fit and then teaching them to work. The aim was to ensure that they did not become a burden on society. Education and training were thus important instruments, as Hutschison has also shown in his study of children at mental asylums in Scotland during the same period.^58^ The syllabus for educable children concentrated on reading and writing, good habits, and preparation for work in practical subjects, such as woodwork or gardening.^59^ Teaching would be based mainly on imitation. Édouard Séguin emphasised that ‘imitation and communication from mind to mind, through language, signs and symbols’ was what was needed.^60^

The teachers at Margarethahemmet were professional educators. The board required from them the ‘determination and the ability to come down to the pupils’ level’, and some were trained at Slagsta skolhem south of Stockholm. Slagsta skolhem was a school for feeble-minded children which also served as a teacher training college.^61^ It provided a two-year course, and included the symptoms of feeble-mindedness and its treatment, and how to teach handicrafts and woodwork, in addition to health care with an emphasis on the central nervous system and speech.^62^ It gave teachers the foundations of both educational methods and practical medical issues. The founders of Slagsta skolhem, especially Thorborg Rappe, had been inspired by leading international educators in the field such as Édouard Séguin and Friedrich Fröbel.^63^ The result was the ‘Slagsta method’, the point of which was to adapt the teaching to the children’s individual learning capacities, both practically and theoretically.^64^ The influence of Séguin is also strong in Rappe’s own lectures. Even the title of her book is almost a direct translation of Séguin’s *Traitement moral, hygiène et éducation des idiots et des autres enfants arriérés ou retardés* (Moral treatment, hygiene and education of idiots and other backward or retarded children).^65^

The teachers at Slagsta skolhem were highly skilled educators and alert to educational trends. They received their education at Slagsta skolhem, which was both an asylum and a teaching centre. The training was equally focused on theoretical and practical elements, based on the argument that for children to be able to learn according to their ability, all parts – heart, brain, and hand – had to be activated.^66^ And the activities were formed in a collaboration between doctors and teachers. Håkan Brockstedt, who has dealt with Slagsta skolhem in his thesis, notes that a distinguishing feature of the education of the so-called ‘abnormal’ in Sweden was that it was believed that only women could become teachers for the feeble-minded.^67^ The reason for this was, as Rappe describes in her lectures, that ‘women’s talents make them more suited to caring for the disadvantaged’.^68^

The circulation of knowledge took place through European networks that influenced professionals in the development of pedagogical methods, but also because Slagsta skolhem played an important role in the training of Nordic special education teachers.^69^ The pedagogical circulation of knowledge is also evident in Rappe’s instructions on nurture and education, where she refers to work from Jean Marc Itard and Carl Wilhelm Saegart, among others.^70^ Rappe’s knowledge of educational developments on the continent and in the United States came from her own study trips to various institutions for the feeble-minded. In addition, she was invited to a conference entitled *Idioten Heilpflege* (Therapeutic care of idiots) in Hamburg 1880.^71^ The conference as well as its journal, *Zeitschrift für die Behandlung Schwachsinniger und Epileptischer* (the Journal for the Treatment of the Feeble-minded and Epileptics), were two ways in which pedagogical knowledge transfer took place. The ideas in the teacher training institute at Slagsta were in line with the international approaches to educating feeble-minded and ‘epileptic’ children.

## Seizures and their consequences

Some of the educable children at Margarethahemmet went on to a life outside the asylum once they had left school, but for others the future held institutionalisation. In fact, in most cases, residents had to obtain a medical certificate to be allowed to leave. An important criterion was not being a burden on society – they had to hold down a job and pay their way.^72^ The ‘uneducable’ residents at Margarethahemmet were expected to help with all sorts of everyday tasks, depending on the severity of their condition.^73^ The rationale was that a calm setting and appropriate treatment would lead to some of those considered uneducable would becoming able to attend lessons alongside the educable.^74^ The question of being educable or uneducable was thus still bound up with the importance of moral education in becoming a self-reliant citizen.

In 1915, Marcus wrote that ‘epilepsy is not one disease, but only a symptom with many different causes’.^75^ Therefore, Marcus believed that a condition for real treatment, leading to a significant improvement for patients with epileptic disorder, was to determine the cause of the disease.^76^ That is, to ascertain what the seizures were due to and the root cause of the disease. Epileptic seizures were a significant obstacle, which made it difficult for the children to attend school properly and regularly. In this context, Henry Marcus’ ideas have been important not only for the actual care and treatment of epilepsy but also regarding the circulation of medical knowledge about epilepsy both amongst those who worked in the asylums and amongst other medical colleagues. Alongside his work at Margarethahemmet, Marcus was a consultant at *Solnas Sjukhem* (Solna’s Nursing Home) for the mentally ill between 1896 and 1937, where new treatment methods were tested, and from 1923 he was also a professor of neurology at *Serafimerlasarettet* (Seraphim Hospital) in Stockholm – Sweden’s first modern hospital.^77^ Thus, for Marcus, children with epilepsy were both patients and subjects of medical research.^78^ Marcus noted that, at Margarethahemmet, children with epilepsy that were classified as feeble-minded were particularly plagued by periods of ‘extremely numerous’ seizures.^79^ During severe, prolonged seizures, Marcus observed in many cases ‘a change in the psychological personality’, with the result that their intellect ‘over time became increasingly limited and sluggish’.^80^ The annual report from 1916 reported that three children had been moved down from the school to the care ward, probably because severe seizures meant they could not cope with the schoolwork.^81^

For the majority of the children deemed uneducable, school classes were not an option. Marcus gave the example of a 7-year-old girl who was diagnosed with ‘idiocy’ – meaning she fell into the group that the German psychiatrist Wilhelm Weygandt defined as ‘feeble-minded, in whom at best the first steps in language development and some possibility of speech recognition can be achieved, but where there can be no question of any inner learning potential’.^82^ Marcus vividly described that the girl had no speech, uttered only lilting palatal sounds, and constantly ground her teeth. According to Marcus, she comprehended nothing and completely lacked intelligence.^83^ She was further described a ‘dirty in the extreme (*hög grad osnygg*) and must be taken care of completely’.^84^ According to psychiatrist Malin Appelquist, who has analysed the care of patients with mental illness around 1900, the term *osnygg* (dirty) was used in medical records to describe the patient’s ability to maintain personal hygiene. Grade 1 indicated that the patient had no bladder control, whereas grade 2 indicated double incontinence. Grade 3 indicated that the patient smeared their faeces, and grade 4 patients were known to eat their faeces.^85^ Marcus’ description as ‘dirty in the extreme’ was probably comparable to grade 3 or 4. Despite the difficulties for this patient and others like her in getting proper medical treatment, Marcus insisted on the need to provide them with good care, i.e. care based on modern science and pedagogy. Marcus believed Margarethahemmet could provide that.^86^ The aim was a good upbringing and the opportunity to teach some basic skills such as hand–eye coordination, manual work such as gardening, and the principle of differentiating between weekdays and weekends.^87^ It involved what the Belgian psycho-pedagogue Ovide Decroly called medico-pedagogy. That is, to cure the children by educating them.^88^ Like many others who work with ‘idiots’ at that time, Decroly was inspired by Pestalozzi, Fröbel, Rousseu, and Dewey, among others.^89^

## Antiepileptics and new diagnostic methods

For a person with epilepsy as well as the ‘feeble-minded’, self-sufficiency was a necessary key to some kind of quality of life.^90^ It was important, therefore, to discover the causes of seizures and develop new medicines. At Margarethahemmet, antiepileptics and new diagnostic methods were put to the test. The most effective antiepileptic drug of the day for both children and adults was bromide.^91^ In many cases, seizures were significantly reduced by bromide therapy, but just as often the seizure frequency returned despite large doses of the drug. The use of bromide persisted, but it also had severe side effects. In 1915 Marcus described that

unfortunately, the results of the bromide treatment undertaken at Margarethahemmet in the past year have been anything but encouraging. A certain reduction in seizures has probably been observed at times in very young and early-stage patients. […] It should be noted that doses often have to be high and that one must prepare for symptoms of ‘bromism’, such as a degree of lethargy and dizziness, poor nutrition, spots, etc. […] … this bromism is a requirement for the bromide to have an effect on epilepsy.^92^

Another possible side effect was impaired motor skills. However, according to the doctors, such side effects were not sufficient reasons to interrupt treatment; this was only considered necessary if the patient developed a high fever, irregular breathing, or became extremely sleepy.^93^ Marcus advocated treatment with opium-bromide, arguing that this treatment usually showed better results.^94^ The same suggestion, that bromide treatment should be preceded by a course of opium, had been discussed among researchers at a medical conference in Berlin in 1897.^95^ Presumably their discussions had then been reported in medical journals that Marcus read, and thus circulation of knowledge was initiated.

When Erik Goldkuhl, Margarethahemmet’s doctor in the 1960s, summarised Marcus’ medical treatment methods, he said he was surprised that phenobarbital, marketed under the trade name Luminal, did not seem to have gained much ground in Sweden in the 1910s.^96^ After all, the German psychiatrist Alfred Hauptmann had introduced it as an antiepileptic drug as early as 1912.^97^ The sources provide no obvious explanation why Luminal was not used at that time, but Goldkuhl surmised it may have been because it was ‘relatively toxic’ and ‘at larger doses for longer periods often resulted in unpleasant chronic poisoning’.^98^ More recent research has also pointed out that Hauptmann published his discovery in a relatively obscure German journal, and that the First World War brought an abrupt hiatus in the circulation of medical knowledge.^99^ Because external circumstances curtailed the possibility of meeting and circulating knowledge, it was not until the 1920s that Luminal’s antiepileptic properties became widely known and used around the world.^100^ In Sweden, it seems to have come into use even later. Henry Marcus first mentions it in 1933 in a lecture in which he describes Luminal as ‘a superb drug, which in small doses keeps the majority of moderately severe cases to within very good limits’.^101^

In the 1920s, the psychiatrist Hans Berger discovered it was possible to use electroencephalograms to register electrical activity in the cerebral cortex in living subjects.^102^ An additional diagnostic method employed in the period was encephalography. Pneumoencephalography was an X-ray examination of the patient’s brain after air had been injected into the fluid-filled cavities to reveal local changes, and it led to diagnoses that had not been possible before.^103^ It was a painful method, and many objections were raised within the medical profession against the use of encephalography. But one of Germany’s leading pioneers in epilepsy surgery, Professor Otfried Foerster, was convinced it was a significant advance in the search to find new diagnostic methods to address the epilepsy problem.^104^ He argued that almost every new method or scientific discovery must count on critical voices and that the criticism was largely because of ‘unfortunate individual cases or the lack of diagnostic benefits in other cases’.^105^

At the general meeting for care of the feeble-minded in Uppsala in 1933, Marcus spoke in positive terms about trials of new treatments and the importance of post-mortem examinations of ‘epileptics’ to gain as much knowledge as possible about the disease.^106^ Among those at Margarethahemmet considered to have idiopathic (also called genuine or authentic) epilepsy – i.e. epilepsy without known damage to the brain from, for example, external violence – Marcus noted that some, upon later examination, were found to have had ‘pathological changes in the brain’ that could have caused the seizures.^107^ Congenital developmental disorders had led many to fear the element of heredity in epilepsy, but according to Marcus, it was important to distinguish between congenital and hereditary epilepsy, if only so ‘epileptics’ could live as normal a life as possible.^108^ Since the seventeenth century, Sweden had banned people with presumed hereditary epilepsy from marrying. As previous research has shown, the marriage ban became increasingly untenable in the twentieth century.^109^ In 1933, Marcus was thinking along the same lines. He believed the lack of knowledge in society about the latest medical findings was a direct cause of the restrictions on people with epilepsy, even though medical science could prove that congenital and hereditary epilepsy were two different issues. Birth defects or events during pregnancy could cause the child to develop epilepsy, but it did not mean the disease was hereditary.^110^ Neither, hypothesised Marcus, was hereditary epilepsy necessarily inherited in direct descent, as it could skip ‘a generation or two, or branch out sideways to manifest there’.^111^ For that reason, he argued it was important there was a post-mortem examination or microscopic examination of the brain to confirm any findings.^112^ A review of contemporary medical articles from Germany shows that Marcus had a good grasp of the latest findings in epilepsy research in Europe. He cited the German neurologist Walter Spielmeyer and his theory of the importance of the hippocampus for the onset of epileptic seizures.^113^ Yet, when citing Spielmeyer, Marcus stated that further investigations were needed to confirm that it was a matter of heredity, and not caused by, say, encephalitis.^114^ Spielmeyer can also be seen as an example of the importance of foreign contacts and knowledge for Swedish health care during that period. After Spielmeyer’s death, Henry Marcus described him as ‘the foremost pioneer in brain research’.^115^ He also mentions Spielmeyer’s regular visits and lectures at the *Svenska Läkaresällskapet* (Swedish Society of Medicine), where he had also held the position of foreign member.^116^

In terms of medical treatment and care, the influence of the United States and the United Kingdom grew over time. In one of Margarethahemmet’s annual reports, for example, Goldkuhl mentioned phenytoin (sold in Sweden as Difhydan) as an important discovery. Phenytoin was first used in the United States in the 1930s to good effect, reducing the frequency of seizures, but it could have strong side effects such as ‘abnormal growth of the gums and sometimes of the lips’, which meant in the end it was used sparingly.^117^ However, many of the children at Margarethahemmet had ‘problems more psychiatric than neurological’.^118^ For these reasons, Goldkuhl still stressed that healthy habits, i.e. a peaceful setting with a clear daily rhythm and routines and ‘the staff’s psychological knowledge, interest, and understanding’ often had greater importance for the care and treatment of the children.^119^

## Knowledge production about epilepsy and its causes

Despite the good care that has been described – with daily routines, nursing, and treatments – every year new children arrived at Margarethahemmet as others were relocated to other care facilities, returned home, or died.^120^ Of the causes of death, one was *status epilepticus*, meaning recurrent, prolonged seizures. Others died of acute pneumonia or tuberculosis.^121^ Tuberculosis was common among residents, and besides the associated deaths, it also meant many of them experienced long periods of fever.^122^ To tackle this, the doctors at Margarethahemmet recommended that patients spend more time outdoors. To make this possible even under bad weather conditions, a play hall was built in the garden and a roof terrace was covered with glass so that even those who were bedridden in the ward could get out into the fresh air.^123^

It was far harder to manage the children’s epileptic seizures. One reason for this was the range of theories and beliefs about what exactly epilepsy was and where in the body it was triggered. Treatment of epilepsy had long consisted of bloodletting, enemas, laxatives, and cauterisation (branding).^124^ Progress was being made, however, especially by doctors interested in medical psychiatry who pursued cures to relieve seizures. By the mid-nineteenth century, the British physician Thomas Laycock had reduced the frequency of seizures among some patients by giving them bromide and performing various kinds of brain surgery.^125^ Another discovery of great significance for epileptic care at the same time was made by John Hughlings Jackson, whose research and clinical studies showed how certain forms of focal epilepsy started, and that the cause of the seizures was linked to discharges in the cerebral cortex.^126^ Despite the medical advances, epilepsy was still a condition that baffled and caused disagreement among doctors in the early twentieth century. As pointed out earlier, Marcus did not consider epilepsy to be a disease as such, but rather a symptom that could be due to various causes.^127^ For that reason, Marcus argued that the objective of medicine was to find a treatment to suit each patient.^128^ Of importance for the treatment was also the international knowledge circulation of epilepsy. Margarethahemmet’s annual reports provide an example of this. In 1926, it was noted that ‘both Margarethahemmet’s medical inspector and board member Professor Marcus and its resident doctor in Knivsta, Dr (Gustaf) Grapengiesser are giving their full attention to the question of surgical intervention for the patients and, alongside it, continuing modern medical treatment.’^129^ During the twentieth century, epilepsy surgery had evolved from being mainly about removing superficial tumours to becoming more highly specialised surgery aiming to eliminate or reduce seizures. However, it was not a harmless method and not everyone who had the operation became seizure-free. To learn more about modern medical treatments, Grapengiesser, Marcus, Herbert Olivecrona, and the previously mentioned pioneer in epilepsy surgery, Professor Otfried Foerster, spent May that year on ‘a neurological examination of a large number of resident children to establish the extent to which modern treatment methods for epilepsy might be applied’.^130^ The Swedish doctors’ contacts abroad thus made it possible for the residents of Margarethahemmet to receive the most up-to-date medical examinations, even though not all of those examinations led to treatment or surgery. It is also an example of circulation of knowledge, e.g. how medical findings about epilepsy came to transcend geographical and institutional boundaries.

## Circulation of treatment and education

This article demonstrates how new research and new knowledge were sought both nationally and internationally to provide doctors and special teachers at the asylum with a proper knowledge about the education, care, and treatment of children with epilepsy. The Swedish epileptic colony Margarethahemmet and its medical inspector Henry Marcus are used as examples.

For much of history, epilepsy was seen as a psychiatric illness and was thus associated with mental health care. The medical approaches to epilepsy in Sweden, and to other psychiatric disorders, were subject to powerful international influence during this period. The majority of references were in the first instance to Germany’s medical publications, and it was mainly from there that Marcus gained his knowledge. However, journals also reported on research in other countries. For example, Otfried Foerster, who visited Margarethahemmet in 1926, kept up to date with the latest medical discoveries as joint editor of the German journal *Zeitschrift für die gesamte Neurologie und Psychiatrie* (Journal of Neurology and Psychiatry Combined) and as a member of several neurological societies in Europe and the United States. His study trips took him to leading research groups abroad. In 1934, supported by the Rockefeller Foundation, Foerster opened a new neurological research institute in his hometown of Breslau, which in time attracted neurosurgeons and neurologists to participate in his research and clinical studies.^131^

The reciprocal process of circulation of knowledge was visible both within Sweden between doctors and teachers in the institutions and between the scientific exchanges – both in medicine and education – that took place through study trips, lectures, and articles disseminated in scientific fields. In a biography from the 1960s, Otfried Foerster and several others, including Hughlings Jackson, were described as having been at the cutting edge of neurology.^132^

Henry Marcus’ research and work at various Stockholm clinics brought him in contact with the leading neurologists of the day, and he also visited Foerster’s clinic. Marcus’ contacts abroad and the study of international scientific journals made it possible for doctors and other staff at the epileptic asylum to keep up to date with the most recent knowledge. The medical inspector for Margarethahemmet hoped that the German professor, Otfried Foerster, could contribute with new expertise and knowledge about surgery. After examining children at Margarethahemmet, Foerster gave his expert opinion on its residents. This led to some of the children being admitted to Seraphim Hospital in Stockholm for observation, although no surgery was attempted. However, the board instructed Marcus to pay a visit to Foerster and his clinic in Breslau in the autumn of 1926 on Margarethahemmet’s behalf, as part of a study tour of clinics abroad.^133^

The collaborations with Margarethahemmet also gave Marcus and his colleagues the opportunity to test new medicines and treatment methods on the children, and to conduct post-mortem examinations of those who died while he was resident there. In 1933 Marcus underlined that patients at the epilepsy colony were ‘a notably rich source for scientific research on epilepsy’.^134^ The main motivation to develop, test, and apply new medicines and methods was to reduce the frequency and intensity of seizures. This was considered essential because the fewer seizures the children had, the more likely they were to be able to learn; and the more educable they were, the easier it was to adapt to society outside the institutions. For this reason, new research and knowledge about special education was sought internationally to provide special teachers with a proper education. The teacher training at Slagsta skolhem, under the leadership of Thorborg Rappe, emerged as an important channel to bring doctors and teachers together in discussions about the importance of special education, which, in addition to the usual teaching skills, also included medical care and specific knowledge of the central nervous system. Teachers at Margarethahemmet also attended training. It is clear from Rappe’s instructions that the pedagogical knowledge had been strongly influenced by the training given to the feeble-minded in Europe and the United States. Particular reference is made to the prominent pioneers such as Séguin, Itard, and Fröbel.

In conclusion, the circulation of knowledge meant that care at Margarethahemmet was to some extend both medical and educational, which is related to the perception of medico-pedagogy at that time. With the help of new medical discoveries, the children had the opportunity to try out new medicines and the teachers at the asylum received medical and educational guidance in their work. The new medical discoveries about epilepsy also meant that the view of children as to some extent educable or at least needing to be stimulated and challenged became more prominent than before. Margarethahemmet became a forerunner of epileptic care in Sweden thanks to Henry Marcus’ contacts both with hospitals in Stockholm and his network abroad. These networks meant that medical knowledge of epilepsy came to transcend geographical and institutional boundaries.

## Footnotes

1

Petteri Pietikäinen , Neurosis and Modernity: The Age of Nervousness in Sweden (Leiden: Brill 2007), 60–1; Malin Appelquist , Insania simplex: Empiriska studier av psykisk sjukdom vid förra sekelskiftet (Lund: Lund University, 2019), 28 .

2 Marcus was the doctor in charge from 1913 to 1916, and then the inspecting doctor until 1941, see Erik Sellin , ‘Tiden efter 1915. Föreningens och hemmets ledning’ in Margarethahemmet 50 år (Uppsala: Appelbergs Boktryckeri AB, 1965), 28 .

3

Hsiu-Fang Hsieh and Sarah E. Shannon , ‘Three Approaches to Qualitative Content Analysis’, Qualitative Health Research, 15, 9 (2005), 1277–8816204405 10.1177/1049732305276687.

4

Johan Östling , ‘Circulation, arenas, and the question for public knowledge. Historiographical currents and analytical frameworks’, History and Theory, 58 (2020), 111–64; Johan Östling et al., Circulation of Knowledge: Explorations in the History of Knowledge (Lund: Nordic Academic Press, 2018).

5

Johan Östling and David Larsson Heidenblad , ‘Cirkulation – ett kunskapshistoriskt nyckelbegrepp’, Historiskt tidskrift, 137, 2 (2017), 270 .

6

David Larsson Heidenblad , Den gröna vändningen: En ny kunskapshistoria om miljöfrågornas genombrott under efterkrigstiden (Sweden: Kriterium, 2021), 22.

7

Philipp Sarasin , ‘Was ist Wissensgeschichte?’, Internationales Archiv für Sozialgeschichte der deutschen Literatur 36, 1 (2011), 164 .

8 ‘Bidrag till Sveriges officiella statistik’, in *Hälso- och sjukvård II, Medicinalstyrelsens underdåniga berättelse angående sinnessjukvården i riket för år 1909* (Stockholm: Nordstedt & Söner, 1911), 10, http://share.scb.se/ov9993/data/historisk%20statistik/BISOS%201851-1917%2FBISOS%20K%20H%C3%A4lso-%20och%20sjukv%C3%A5rden1861-1910%2FBISOS%20K%20Sinnessjukv%C3%A5rd%201861-1910%2FHalso--och-sjukvarden-K2-1909.pdf, accessed 5 January 2024.

9

Thomas Barow , ‘Begreppet “obildbar” som en social konstruktion: Teoretiskt diskussion och praktisk tillämpning inom den svenska sinnesslövården under 1900-talets första hälft’, in Olov Andersson , Thomas Barow and Magnus Tideman (eds.), Omsorg i förändring: En vänbok till Karl Grunewald (Stockholm: Intra, 2011), 160 .

10 Cf. Ulrike Beyer , *Die* Versorgung der Epileptiker in Berlin am Beispiel der ‘Anstalt für Epileptische Wuhlgarten bei Biesdorf’ *(unpub.)* (Berlin: Universitätmedizin Berlin, 2017), 13 .

11 For a similar discussion of the use of historical terms, see Beyer, *op. cit.* (note 10), 13; Annemike van Drenth , ‘Doctors, philanthropists and teachers as ‘true’ ventriloquists? Introduction to a special issue on the history of special education’, History of Education, 34, 2 (2005), 107–8.

12

Owsei Temkin , The Falling Sickness: A History of Epilepsy from the Greeks to the Beginning of Modern Neurology (Baltimore: John Hopkins University Press, 1971) (first pub. 1945); Walter J. Friedlander , The History of Modern Epilepsy: The Beginning, 1865-1914 (Westport, CT: Greenwood, 2001).

13

German E. Berrios and Roy Porter (eds.), A history of clinical psychiatry: The origin of psychiatric disorders (London: Athlone, 1995); Lene Koch , Tvangssterilisation i Danmark 1929-67 (Copenhagen: Gyldendal, 2000); Johanna Schoen , Choice and coercion: Birth control, sterilization and abortion in public health and welfare (Chapel Hill: University of Carolina Press, 2005).

14

Peter Wolf , ‘History of epilepsy: Nosological concepts and classification’, Epileptic Disorder, 16, 3 (2014), 261–910.1684/epd.2014.067625256654; Dieter Schmidt and Simon Shorvon , The End of Epilepsy? A History of the Modern Era of Epilepsy Research, 1860-2010 (Oxford: OUP, 2016); Karl Otto Nakken , Fra epilepsikoloni til spesialsykehus (Sandvika: Specialsykehuset for epilepsi, 2006); Scott E. Lipson , Jennifer A. Montes and Orrin Devinsky , ‘Epilepsy in *The Alienist and Neurologist*, 1880-1920’, Epilepsia, 43, 8 (2002), 912–1912181011 10.1046/j.1528-1157.2002.34201.x.

15

Carl Henry Alström , A study of epilepsy in its clinical, social and genetic aspects (Copenhagen: Munksgaard, 1950)14846690; Arvid Carlsson and Lena Carlsson , Hjärnans budbärare (Lund: Studentlitteratur, 2001); Torsten Svenson Frey , Electroencephalographic Study of Neuropsychiatric Disorders: Covering 159 Cases of Epilepsy, 53 Suspected Epilepsy, 122 of Nonepileptic Abnormality and 101 Control Cases (Stockholm: Karolinska institutet, 1946).

16

Gunnar Broberg and Mattias Tydén , Oönskade i folkhemmet. Rashygien och sterilisering i Sverige (Uppsala: Gidlunds, 1991); Mattias Tydén , Från politik till praktik: De svenska steriliseringslagarna 1935-1975 (Stockholm: Almqvist & Wicksell International, 2002).

17

Elin Bommenel , Sockerförsöket: Kariesexperimenten 1943-1960 på Vipeholms sjukhus för sinnesslöa (Lund: Arkiv, 2006); Kristina Engwall , ‘Starved to Death? Nutrition in Asylum During the World Wars’, Scandinavian Journal of Disability Research, 7 (2005), 2–22 .

18

Jona T Garz , Zwischen Anstalt und Schule Eine Wissensgeschichte der Erziehung schwachsinniger Kinder in Berlin 1845-1914, Bielefeld: Transcript, 2022 , Chap. 4; Jona T Garz , ‘Fabricating spaces and knowledge: the Berlin-Dalldorf Municipal Asylum for “Feeble-Minded” Children (1880-1900)’, History of Education Review, 50, 2 (2021), 146–65.

19 Beyer, *op. cit.* (note 10).

20

Margarete Pfäfflin , ‘Bethel, Bielefeld, Germany: Epilepsiezentrum Bethel’, Seizure 12, 2003, 4–8 12547177; Philip Grewe and Christian G Bien , ‘150th anniversary of the Bethel epilepsy center in Germany: An important milestone in the evolution of epilepsy care’, Seizure 53, 2017, 110–329173941 10.1016/j.seizure.2017.11.007; Bjørn Hamre , Syke må plejes med kaerlighed. Kolonien Filadelfia 1897-1997 (Dianalund: Kolonien Filadelfia, 1997).

21

Lena Lennerhed , ‘Annorlunda Nyheter: Patientröster på 1970-talets Stora Sköndal’, in Andres Brink Pinto , Mikael Mery Karlsson and Irina Schmitt (eds.), Kritiska blickar från marginalen: Reflektioner i spåren av Jens Rydström (Lund: Lunds universitet, 2022), 199–218 .

22

Hansjörg Schneble , Heillos, heilig, heilbar: Die Geschichte der Epilepsie von den Anfängen bis heute (Berlin: De Gruyter, 2003), 137 ; Lena Lennerhed , ‘Epileptikern’, in Peter Josephson and Leif Runefeldt (eds.), Historiska typer (Möklinta: Gidlunds, 2020), 127–8, 134-7.

23

Johanna Ringarp , ‘“Nytt ljus över epilepsien”. Den medicinska utvecklingens påverkan på sjukdomen under 1950-talet’, in Anders Burman and Bosse Holmqvist (eds.), Det lyckliga femtiotalet: Sexualitet, politik och motstånd (Stockholm: Symposion, 2019), 114–17; Lena Lennerhed and Johanna Ringarp , ‘Den farliga epileptikern: äktenskapshinder och yrkeshinder i det svenska 1900-talet’, in Motzi Eklöf (ed.), Medicinska moraler och skandaler. Vetenskapens (etiska) gränser (Stockholm: Carlssons förlag, 2019), 138–159 ; Ann Jacoby , ‘Stigma, epilepsy, and quality of life’, Epilepsy & Behavior, 3 (2002), 10 10.1016/s1525-5050(02)00545-012609301.

24 International Bureau for Epilepsy, ‘What is epilepsy?’, https://www.ibe-epilepsy.org/what-is-epilepsy-2, accessed 5 January 2024.

25 The same applied in Germany, see Beyer, *op. cit.* (note 10), 9, 13, 15.

26

Judith Areschoug , Det sinnesslöa skolbarnet: Undervisning, tvång och medborgarskap 1925-1954 (Linköping: Linköping universitet, 2000), 53–7, 62-3; Mårten Söder, *Anstalter för utvecklingsstörda: En historisk–sociologisk beskrivning av utvecklingen* (Stockholm: Arla, 1984) (first pub. 1978), 6-8, 62-7.

27 Areschoug, *op. cit.* (note 26), 64-7; Marja Runcis , Makten över barnen: Tvångsomhändertaganden av barn i Sverige 1928-1968 (Stockholm: Atlas, 2007), 39–42 , 45-8.

28
*Föreningen Margarethahemmet för vård af fallande sjuka sinnesslöa barn 1890-1915* (Stockholm: Centraltryckeriet 1915), 11; Schneble, *op. cit.* (note 22), 105; Erik Goldkuhl , ‘Epileptikervården’, in Margarethahemmet 50 år (Uppsala: Appelbergs Boktryckeri AB, 1965), 51 .

29

Ulf-Johan Olsson , Det räcker inte med kunskap om epilepsi… Epilepsivården för vuxna ur ett kunskapsstyrnings- och patientperspektiv (Stockholm: Svenska Epilepsiförbundet 2009), 18 .

30

Sara Hansson , I den goda vårdens namn: sinnesslövård i 1950-talets Sverige (Uppsala: Uppsala universitet, 2007), 45 .

31 Ringarp, *op. cit.* (note 23), 113-16.

32

SOU 1955:52, Epileptikervården. Förslag av Medicinalstyrelsens delegation för epileptikervård (Stockholm: Inrikesdepartementet, 1955), 18 .

33 Areschoug, *op. cit.* (note 26), 68.

34 Goldkuhl, *op. cit* (note 28), 51.

35

Ivar Törnebladh and Immanuel Kiellman-Göransson , ‘Rapport från hemmet för sinnesslöa och af fallandesjuka lidande barns vård’, in Föreningen för vård af fallandesjuka och sinnesslöa barnhemmet vid Mariehäll berättelse för 1892 (Stockholm 1893), 7–8 .

36

Owe Røren , Idioternas tid. Tankestilar inom den tidiga idiotskolan 1840-1872 (Stockholm: Stockholm University, 2007), 81–3.

37
*Margaretahemmets årsberättelse för år 1907* (Stockholm: Gustafson & Påhlsons boktryckeri, 1908), 9.

38
*Margaretahemmets årsberättelse för år 1907*, *op. cit.* (note 37), 9.

39 Sellin, *op. cit.* (note 2), 14; Erik Sellin ,‘Egendomen’ in Margarethahemmet 50 år (Uppsala: Appelbergs Boktryckeri AB, 1965), 33 .

40 Sellin, *op. cit.* (note 2), 13-4.

41
*Margaretahemmets årsberättelse för år 1907*, *op. cit.* (note 37), 8-9.

42
*Föreningen Margarethahemmet*, ‘Tiden 1916-1969’, http://www.margarethahemmet.se/Home/Tiden_1916__1969, accessed 5 January 2024.

43

Alfred Petrén ,‘Bör sinnesslövården göras obligatorisk?’, in Förhandlingar vid sjätte allmänna svenska mötet för vården om de sinnesslöa hållet i Stockholm 10-12 juni 1915 (Stockholm: Oskars Eklunds boktryckeri, 1915), 112 ; Thorborg Rappe , Några råd och anvisningar vid sinnesslöa barns (idioters) vård, uppfostran och undervisning (Stockholm: O. von Feilitzen, 1903), 14; Iain Hutchison , ‘Institutionalization of mentally-impaired children in Scotland, c. 1855-1914’, History of Psychiatry 22, 4 (2011), 424 10.1177/0957154X1039043622530371.

44

Bjørn Hamre , Thom Axelsson and Kari Ludvigsen , ‘Psychiatry in the sorting of schoolchildren in Scandinavia 1920-1950 – IQ testing, child guidance clinics and hospitalization’, Paedagogica Historica: International Journal of the History of Education 55, 3 (2019), 396 .

45

Henry Marcus ,‘Epilepsi och sinnesslöhet: Några ord vid Margarethahemmets invigning’, in Föreningen Margarethahemmet för vård af fallandesjuka sinnesslöa barn 1890-1915 (Stockholm: Centraltryckeriet 1915), 75 .

46 Marcus, *op. cit.* (note 45), 75.

47

Alfred Petrén , ‘Riktlinjer för epileptikervårdens utveckling i vårt land’, in Förhandlingar vid tolfte allmänna svenska mötet för vården om de sinnesslöa hållet i Bollnäs den 20-21 juni 1938 (Eslöv: Tryckeribolaget, 1939), 42–3.

48 Hansson, *op. cit.* (note 30), 12.

49

Erik Ekelöf , ‘Läkarrapport från Margarethahemmet för år 1916’, in Margaretahemmets årsberättelse för år 1916 (Stockholm: Beckmans boktryckeri, 1917), 21–2.

50
*Margaretahemmets årsberättelse för år 1924* (Stockholm: Beckmans boktryckeri, 1925), 3.

51
*Margaretahemmets årsberättelse för år 1939* (Stockholm: Beckmans boktryckeri, 1940), 3.

52

Jonas Jonsson ,‘Grad af idiotism hos de intagna och i samband därmed stående förmåga af bildbarhet’, in Föreningen för vård af fallandesjuka och sinnesslöa barnhemmet vid Mariehäll berättelse för 1892 (Stockholm: Gustafson & Påhlsons boktryckeri 1893), 7–8 .

53

Carl Sebardt , ‘Epileptikervård: En historik över epileptikervården i Sverige, speciellt om densamma å Stora Sköndal under 25-årsperioden 1905-1930’, Hygiea 23, 92 (1930), 884 .

54
*Föreningen Margarethahemmet …*, *op. cit.* (note 28), 5-9.

55

Barbara Kuhlo , ‘Der kindliche Schwachsinn als diagnostisches, therapeutisches und sociales Problem im 19. Jahrhundret’, Medizinhistorisches Journal 9, 2 (1974), 127 11609313. Cf. Hamre, *op. cit.* (note 20), 14, 26-7.

56

Annemieke van Drenth , ‘Mental Boundaries and Medico-pedagogical Selection: Girls and Boys in the Dutch ‘School for Idiots’, The Hague 1857-1873, Paedagogica Historica 43, 1 (2007), 106 .

57

Håkan Brockstedt , Slagsta skola och seminarium och dess föregångare: skolan för sinnesslöa barn i Stockholm: ideologi och praktik i undervisningen av barn med utvecklingsstörning 1870-1950 (Stockholm: Stockholm universitet, 2000), 11–15 ; Garz*, op. cit.* (note 18), 15-18.

58 Hutchison, *op. cit.* (note 43), 424.

59

Ingrid Fröstedt , ‘Moderna undervisningsidéer tillämpande i sinnesslöskolan’, in Förhandlingar vid elfte allmänna svenska mötet för vården om de sinnesslöa i Uppsala den 15-16 juni 1933 (Uppsala: Wretmans boktryckeri, 1933) 85–95 .

60

Èdouard Séguin , Idiocy and its treatment by the physiological method (New York; William Wood & Co, 1866), 33–4, https://iiif.wellcomecollection.org/pdf/b21292425, accessed 3 January 2024

61

Viola Karlsson ,‘Undervisningen’, in Margarethahemmet 50 år (Uppsala: Appelbergs Boktryckeri AB, 1965), 64 , 67, quote at 67; Brockstedt, *op. cit.* (note 57), 32-3.

62

Johan Ambrosius , ‘Lärarebildning för sinnesslöanstalterna’, in Förhandlingar vid sjunde allmänna svenska mötet för vården om de sinnesslöa hållet i Mariestad 20-22 juni 1916 (Mariestad: Länstidningens tryckeri, 1916), 46–7.

63 Røren, *op. cit.* (note 36), 119-21.

64 Barow, *op. cit.* (note 9), 152, 380; Owe Røren, ‘Thorborg Ragnhild Rappe’, *Svenskt Kvinnobiografiskt Lexikon*, www.skbl.se, s.v. ‘Thorborg Rappe’, accessed 5 January 2024.

65 Brockstedt, *op. cit.* (note 57), 77.

66 Brockstedt, *op. cit.* (note 57), 77. Cf. van Drenth, *op. cit.* (note 11), 110.

67 Brockstedt, *op. cit.* (note 57), 169-77.

68 Rappe, *op. cit.* (note 43), 39.

69 Brockstedt, *op. cit.* (note 57), 200; Birgitta Salin , Utmaning och omtanke. En analys av handling som en utvidgad specialpedagogisk funktion i skolan med utgångspunkt i tio pionjärers berättelser (Stockholm: HLS Förlag, 2006), 30–1.

70 Rappe, *op. cit.* (note 43) 9-12.

71

E. Reichelt , Bericht über die IV. Konferenz für Idioten-Heil-Pflege Hamburg (Dresden: Qarnatz & Lehmann 1883), 4 .

72 Areschoug, *op. cit.* (note 26), 225.

73 Karlsson, *op. cit.* (note 61) 65-7; cf. *Reglemente för elevers intagning i skolan för sinnesslöa barn i Stockholm* (Uppsala: Ed Berling, 1876), 1.

74

Jonas Jonsson , ‘Läkarrapport från sjukhemmet Mariehäll för år 1893’, in Föreningen för vård af Fallandesjuka sinnesslöa barn. Hemmet vid Mariehäll, styrelsens berättelse för 1893 (Stockholm: Gustafson & Påhlsons boktryckeri 1894), 11 .

75 Marcus, *op. cit.* (note 45), 90.

76 Marcus, *op. cit.* (note 45), 88.

77 Pietikäinen, *op. cit.* (note 1), 105-106; Lars Öberg, ‘Henry I Marcus’, *Svenskt Biografiskt Lexikon*, sok.riksarkivet.se/sbl/, ‘Henry Marcus’, accessed 5 January 2024.

78 Cf. Cecilia Riving , Icke som en annan människa: Psykisk sjukdom i mötet mellan psykiatrin och lokalsamhället under 1800-talets andra hälft (Hedemora: Gidlunds, 2008), 285–6.

79 Marcus, *op. cit.* (note 45), 73. However, it is not clear in the report how long such a period of symptoms could be.

80 Marcus, *op. cit.* (note 45), 74.

81 Ekelöf, *op. cit.* (note 49), 21-2.

82 Marcus, *op. cit.* (note 45), 77.

83 Marcus, *op. cit.* (note 45), 77.

84 Marcus, *op. cit.* (note 45), 77.

85 Appelquist, *op. cit.* (note 1), 97-8.

86 Marcus, *op. cit.* (note 45), 80-1, 94. Cf. Areschoug, *op. cit.* (note 26), 62-7.

87 Marcus, *op. cit.* (note 45), 79.

88

Angelo van Gorp , ‘From special to new education: the biological, psychological, and sociological foundation of Ovid Decroly’s educational work (1871-1932)’, History of Education 34,2 (2005), 135 ; Paolo Bianchini , ‘The ‘Meidco-Pedagogical Institutes’ and the failure of the collaboration between psychiatry and pedagogy (1889-1978)’, Paedagogica Historica 55, 3 (2019), 511–27; van Drenth, *op. cit.* (note 56), 116.

89 van Gorp, *op. cit.* (note 88), 135-49. Cf. Røren, *op. cit.* (note 36), 85-90.

90 Ringarp, *op. cit.* (note 23), 116-18.

91

Emmanouil Magiorkinis et al., ‘Highlights in the History of Epilepsy: The Last 200 Years’, Epilepsy Research & Treatment (2014), 5 10.1155/2014/582039PMC415825725210626; Schneble, *op. cit.* (note 22), 104, 124.

92 Marcus, *op. cit.* (note 45), 91.

93

Wolfgang Warda , ‘Erfahrungen über die Behandlung der Epilepsie mit Opium-Brom’, Monatsschrift für Psychiatrie & Neurologie, 2/4 (1897), 259 .

94 Marcus, *op. cit.* (note 45), 91.

95

Paul Flechsig , ‘Discussion’, in Ernst von Leyden and Emil Pfeiffer (eds.), Verhandlungen des Congresses für Innere Medicin Fünfzehnter Congress, Gehalten zu Berlin vom 9-12 Juni 1897 (Wiesbaden: Verlag von J. F Bergmann, 1897), 192 ; Theodor M. Rumpf , ‘Discussion’, Verhandlungen des Congresses für Innere Medicin Fünfzehnter Congress, Gehalten zu Berlin vom 9-12 Juni 1897 (Wiesbaden: Verlag von J. F Bergmann, 1897), 199 .

96 Goldkuhl, *op. cit.* (note 28), 52.

97

Hansjörg Schneble , ‘Aus der Geschichte der Epilepsie’, Zeitschrift für Epileptologie, 27, 208 (2014), 208 ; Magiorkinis et al., *op. cit.* (note 91), 28.

98 Goldkuhl, *op. cit.* (note 28), 53.

99 Karl Otto Nakken and Eylert Brodtkorb, ‘Slump, tilfeldigheter og antieepileptika’, *Tidsskrift for den norske Legeforeningen*, 30 October 2017, https://tidsskriftet.no/2017/10/medisinsk-historie/slump-tilfeldigheter-og-antiepileptika, accessed 5 January 2024; Zeid Yasiry and Simon D. Shorvon , ‘How phenobarbital revolutionized epilepsy therapy: The story of phenobarbital therapy in epilepsy in the last 100 years’, Epilpesia, 53, 8 (2012), 27–8 10.1111/epi.1202623205960.

100 Yasiry and Shorvon, *op. cit.* (note 99), 28-30.

101

Henry Marcus , ‘Om den s. k. medfödda epilepsin’, in Förhandlingar vid elfte allmänna svenska mötet för vården om de sinnesslöa hållet i Uppsala den 15-16 juni (Uppsala: Wretmans boktryckeri, 1934), 113 .

102

Hans Berger , ‘Über das Elektrenkephalogramm des Menschen’, *M* ittteilung, Archiv für Psychiatrie & Nervenkrankheiten, 87, (1929), 527 ; cf. Mehdi Laghmari et al., ‘The epic history of epilepsy surgery’, North African & Middle East Epilepsy Journal, 1, 6 (2012), 28 ; James L. Stone and John R. Hughes , ‘Early history of electroencephalography and establishment of the American Clinical Neurophysiology Society’, Journal of Clinical Neurophysiology, 30, 1 (2013), 29.10.1097/WNP.0b013e31827edb2d23377440

103

Theodor Bársony and Ladislaus Balassa , ‘Über Röntgenbestrahlung des Schädels bei Epilepsie’, Zeitschrift für die gesamte Neurologie & Psychiatrie, 113, (1928), 757–60; William Feindel , Richard Leblanc and Antonio Nogueira de Almeida , ‘Epilepsy Surgery: History Highlights 1909-2009’, Epilepsia, 50, (2009), 136–710.1111/j.1528-1167.2009.02043.x19298436; Robert Gaupp , ‘Otfried Foerster: Sein Lebenswerk im Rahmen der Wissenschaft seiner Zeit’, Zeitschrift für die gesamte Neurologie & Psychiatrie, 176, (1943), 502–3; Cf. Hamre, *op. cit.* (note 20), 138.

104 Gaupp, *op. cit.* (note 103), 503-4.

105

Otfried Foerster , ‘Encephalographische Erfahrungen’, Zeitschrift für die gesamte Neurologie & Psychiatrie, 12, 94 (1925), 512 .

106 Marcus, *op. cit.* (note 101), 109, 113.

107 Marcus, *op. cit.* (note 101), 108.

108 Marcus, *op. cit.* (note 101), 108.

109 Lennerhed and Ringarp, *op. cit.* (note 23), 139-46.

110 Marcus, *op. cit.* (note 101), 112-3.

111 Marcus, *op. cit.* (note 101), 109.

112 Marcus, *op. cit.* (note 101), 109.

113

Walter Spielmeyer , ‘Der Gegenwärtige Stand der Epilepsieforschung: III Teil Anatomisches’, Zeitschrift für die gesamte Neurologie & Psychiatrie, 89, (1924), 363 , 366.

114 Marcus, *op. cit.* (note 101), 109; cf. Spielmeyer, *op. cit.* (note 113), 367.

115 Henry Marcus, *Walter Spielmeyer. Minnesord i Svenska läkaresällskapet* (Stockholm, 1935) 161.

116 Marcus, *op. cit.* (note 115), 161-70.

117 Goldkuhl, *op. cit.* (note 28), 53.

118 Goldkuhl, *op. cit.* (note 28), 55.

119 Goldkuhl, *op. cit.* (note 28), 55.

120 Ekelöf, *op. cit.* (note 49), 21-2; ‘Tabell över vid Margarethahemmet befintliga patienter år 1926’, in *Margaretahemmets årsberättelse för år 1926* (Stockholm: Beckmans boktryckeri, 1927), 4-5; ‘Tabell över vid Margarethahemmet befintliga patienter år 1939’, in *Margaretahemmets årsberättelse för år 1939* (Stockholm: Beckmans boktryckeri, 1940) 4-5.

121 Ekelöf, *op. cit.* (note 49), 21-2; Gustaf Grapengiesser , ‘Läkarberättelse för år 1926’, in Margaretahemmets årsberättelse för år 1926 (Stockholm: Beckmans boktryckeri, 1927), 19 .

122 Cf. Barow *op. cit.* (note 9), 161-2.

123 Grapengiesser, *op. cit.* (note 121), 19.

124 Nils Antoni, *Nervsjukdomar* (Stockholm: Wahlström & Widstrand, 1928); Bardia Tajerbashi & Christoph Friedrich , ‘Zur Geschichte der Therapie der Epilepsie: Eine der ältesten bekannten Krankheit’, Pharmazie in unserer Zeit, 36, 4 (2007), 254 17623314 10.1002/pauz.200600223.

125 Antoni, *op. cit.* (note 124); Eugen Kapelusch , *‘Discussion’*, in Verhandlungen des Congresses für Innere Medicin Fünfzehnter Congress, Gehalten zu Berlin dem 9-12 Juni 1897, eds. Ernst von Leyden and Emil Pfeiffer (Wiesbaden: Verlag von J. F Bergmann, 1897), 198–9.

126

Claus-Werner Wallesch , ‘Huglings Jackson and European Neurology’, in Hierarchies in Neurology, eds. Christopher Kennard and Michael Swash (Berlin & Heidelberg: Springer, 1989) 17–23 ; Michael R. Trimble , ‘Psychiatric Aspects of Temporal Lobe Epilepsy’, in Hierarchies in Neurology, eds. Christopher Kennard and Michael Swash (Berlin & Heidelberg: Springer, 1989) 59–63 ; George K. York and David A. Steinberg , ‘Hughlings Jackson’s neurological ideas’, Brain, 134 (2011)10.1093/brain/awr21921903729, quote at 3106.

127 Marcus, *op. cit.* (note 45), 90.

128 Marcus, *op. cit.* (note 45), 90.

129
*Margaretahemmets årsberättelse för år 1926, op. cit* (note 120), 8.

130 Grapengiesser, *op. cit.* (note 121), 19.

131 Gaupp, *op. cit.* (note 103), 487–8, 506, 509; Levent Sarikcioglu , ‘Otfried Foerster (1873-1941): One of the distinguished neuroscientists of his time’, Journal Neurology Neurosurgery & Psychiatry, 78, 6 (2007), 650 10.1136/jnnp.2006.112680PMC207795717507449; Schneble, *op. cit.* (note 22), 128.

132

Klaus Joachim Zülch , ‘Biography of Otfrid Foerster’, in ‘Otfried Foerster: Physician and Naturalist, November 9, 1873-Juni 15, 1941, ed. Klaus Joachim Zülch (Berlin & Heidelberg: Springer, 1969), 12 .

133
*Margaretahemmets årsberättelse för år 1926*, *op. cit.* (note 120), 8.

134 Marcus, *op. cit.* (note 101), 107.

